# The power of loss: message framing, climate anxiety, and engagement in personal carbon trading

**DOI:** 10.1186/s40359-025-03713-w

**Published:** 2025-12-29

**Authors:** Tong Li, Yutao Yang, Xiaofei Liu, Siyu Zeng

**Affiliations:** 1 Department of Economics and Trade Management, Shanxi Institute of Mechanical & Electrical Engineering, No. 130, Baoningmen East Street, Changzhi, Shanxi Province 046000 China; 2 Hilton School of Hospitality Management, Sichuan Tourism University, No.459, Hongling Road, Chengdu, Sichuan Province 610100 China; 3 Business School, Sichuan University, No. 24, South Section 1, First Ring Road, Chengdu, Chengdu, Sichuan Province 610065 China; 4 School of Logistics, Chengdu University of Information Technology, No.10, Xingfu Road, Chengdu, Sichuan Province 610103 China

**Keywords:** Personal carbon trading, Message framing, Climate change anxiety, Voluntary environmental behavioral intention

## Abstract

As global carbon emissions continue to rise and climate targets grow increasingly urgent, promoting voluntary low-carbon behaviors has become a critical component of climate policy. Among various behavior-oriented instruments, Personal Carbon Trading (PCT) offers a promising yet underutilized strategy for incentivizing individual emissions reduction, though public participation remains limited due to psychological and communicative barriers. This study investigates how message framing (gain vs. loss) influences individuals’ willingness to participate in PCT, and whether this effect is mediated by climate change anxiety (CCA) and moderated by two psychological traits: felt responsibility (FR) and green self-efficacy (GSE). Drawing upon Prospect Theory, Norm Activation Theory, and Self-Efficacy Theory, we conducted an online experiment with randomized stimuli and analyzed the responses of 788 participants. The results show that loss-framed messages significantly enhance voluntary engagement intention (VEI) towards PCT compared to gain-framed messages. Moreover, CCA partially mediates the relationship between message framing and VEI, confirming the emotional pathway through which message framing operates. Importantly, both FR and GSE moderate these effects, such that loss framing is more effective among individuals with higher levels of responsibility or efficacy. These findings advance theoretical understanding of the framing-emotion-intention pathway while offering evidence-based strategies for designing personalized environmental communications that promote voluntary participation in PCT.

**Supplementary Information:**

The online version contains supplementary material available at 10.1186/s40359-025-03713-w.

## Introduction

Global carbon emissions have reached unprecedented levels, with projections indicating a total of 41.6 billion metric tons by the end of 2024—a 2.5% increase from the previous year’s 40.6 billion metric tons [1]. This surge underscores the escalating challenge of mitigating climate change. The Intergovernmental Panel on Climate Change (IPCC) emphasizes that to limit global warming to 1.5 °C, it is imperative for global greenhouse gas emissions to peak before 2025 and decrease by 43% by 2030. However, current emission reduction efforts fall substantially short of these critical targets. Closing this mitigation gap requires coordinated and immediate action across multiple levels—governments, businesses, and individuals—to implement effective strategies and accelerate the transition toward a low-carbon future^1^.

Governments worldwide have actively implemented environmental policies to mitigate carbon emissions. China, for example, has issued environmental policies including *the Peak Carbon Action Program by 2030*, *the Pilot Policy on Carbon Emissions Trading (2011)*, *the Action Program for Energy Efficiency and Carbon Reduction 2024–2025*,* and the Pilot Policy on Low-Carbon Cities (2010)*. The carbon trading mechanism, as a crucial market-based tool to address climate change, internalizes the cost of carbon emissions through price mechanisms, thereby guiding enterprises, governments, and the public toward low-carbon transitions. However, the effectiveness of carbon trading mechanisms is influenced by various behavioral factors, including policy design, corporate actions, and public participation [2].

In recent years, the growing share of household-related emissions in global carbon output has brought increasing attention to the role of individual behaviors in climate mitigation [3]. Households are estimated to account for approximately 72% of global carbon emissions, with residential energy consumption representing the largest end-use sector [4, 5]. This shift has led researchers to emphasize the necessity of engaging individuals in achieving national and international climate targets [6]. Personal Carbon Trading (PCT), as a behavior-oriented policy instrument, is designed to incentivize low-carbon lifestyles by allocating tradable emission allowances at the individual level. However, despite its conceptual appeal, PCT is often viewed as a relatively radical policy approach and faces considerable challenges in garnering public support and participation [7].

Existing studies have shown that public acceptance of PCT schemes is primarily shaped by factors such as policy design, perceived fairness, and environmental effectiveness [8]. While PCT participation represents a type of pro-environmental behavior, it differs from traditional environmental actions in three fundamental ways. First, PCT operates as a market-based mechanism involving economic transactions rather than purely altruistic actions, potentially creating conflicts between environmental values and economic rationality [9]. Second, it requires understanding complex policy instruments, thereby imposing higher cognitive demands than conventional green behaviors [10, 11]. Third, PCT involves a dual-layered feedback structure: immediate economic feedback through market transactions versus delayed collective environmental outcomes. This creates psychological tension between tangible financial signals and abstract climate goals—a complexity absent in purely altruistic behaviors like recycling or donating [12]. These features directly inform our theoretical framework. The economic-environmental trade-off aligns with Prospect Theory’s loss aversion, suggesting loss-framed messages may be more effective. The cognitive complexity indicates that felt responsibility and green self-efficacy moderate message processing. The dual feedback structure necessitates emotional mechanisms—specifically, climate anxiety—to bridge the gap between immediate economic signals and distant environmental consequences.

Despite extensive pro-environmental behavior research, three gaps remain in the PCT context. First, message framing effectiveness in market-oriented environmental instruments remains underexplored. Second, existing PCT literature emphasizes cognitive pathways [13, 14]. while neglecting emotional mechanisms—particularly whether climate anxiety can motivate voluntary participation. Third, personalized communication strategies that account for psychological trait, such as felt responsibility (FR) and green self-efficacy (GSE) are largely absent. This study addresses these gaps by investigating: (1) whether loss-framed messages outperform gain-framed messages in PCT promotion, (2) whether climate change anxiety (CCA) mediates this relationship, and (3) how individual psychological traits moderate these effects.

This study adopts an online experimental design to investigate how message framing (positive vs. negative) influences individuals’ engagement in PCT. Additionally, this study explores potential heterogeneity across participants, examining whether psychological traits—particularly in GSE and FR—moderate the impact of framing on behavioral intention.

Against the backdrop of global efforts to achieve carbon neutrality, this study offers both theoretical and practical contributions to the growing literature on climate communication and behavioral interventions. Theoretically, drawing upon Prospect Theory, Norm Activation Theory, and Self-Efficacy Theory as complementary foundations, we identify a novel psychological mechanism through which message framing influences PCT participation. Our findings not only confirm the superior persuasive power of loss-framed messages in promoting PCT, but also uncover the mediating role of CCA and the moderating effects of individual psychological traits such as FR and GSE. Practically, this study provides targeted strategies for designing framing-based communication tailored to different audience segments, emphasizing the importance of balancing environmental threat with action efficacy. We also highlight the potential of digital platforms in delivering adaptive, personalized environmental messaging. By revealing the nuanced mechanisms through which message framing interacts with psychological variables to shape the low-carbon behavioral intention, this research contributes valuable insights for both academic inquiry and policy implementation in the field of sustainable intention change.

## Theoretical background and hypotheses development

### Message framing effect

Message framing refers to the use of positive or negative expressions to describe the same information in order to evoke different psychological and behavioral responses [15, 16]. Gain framing emphasizes the positive outcomes of engaging in a behavior. This type of framing highlights the benefits associated with action. Loss framing stresses the negative consequences of not engaging in a behavior. Loss framing is often used to invoke concern or a sense of urgency. In behavioral and consumer research, message framing has been recognized as a critical mechanism for influencing environmental decision-making [17]. According to Prospect Theory, individuals demonstrate loss aversion—that is, they are generally more motivated to avoid losses than to achieve equivalent gains [18]. Empirical studies in environmental psychology have consistently supported the applicability of Prospect Theory, demonstrating that loss-framed messages are generally more persuasive than gain-framed messages in encouraging pro-environmental behaviors. For instance, statements such as “failing to act will worsen climate change” tend to elicit stronger emotional and motivational responses than gain-framed messages like “taking action will help the environment [19].” Loss-framed message has proven particularly effective in promoting specific sustainable behaviors, including recycling, energy conservation, and public support for anti-pollution measures [20–22]. Drawing upon this body of evidence, we argue that in the context of voluntary PCT—a domain involving both environmental responsibility and individual autonomy—loss-framed messaging is more likely to enhance consumers’ behavioral intentions. Accordingly, we propose the following hypothesis:

*H1: The loss-framed message will be more effective than the gain-framed message in promoting voluntary engagement intention (VEI) toward PCT.*

### The mediating role of climate change anxiety

Message framing operates as a strategic communication tool whose persuasive power depends on both message content and recipients’ cognitive-emotional processing [23]. Beyond shaping behavioral decisions, framing systematically influences emotional responses, particularly risk perceptions and affective states such as worry and fear [15]. Anxiety, in this context, is considered an adaptive response to perceived threat, functioning to enhance attentional focus and motivate protective actions [24].

In recent years, the concept of CCA—a psychological state characterized by concern, helplessness, and distress about the future impacts of climate change—has garnered increasing scholarly attention [25]. Importantly, the literature reveals ambivalent effects of climate anxiety. While moderate anxiety can motivate pro-environmental intentions and behaviors [24], excessive anxiety may trigger avoidance, helplessness, or psychological disengagement [26, 27]. This dual nature suggests anxiety’s effects depend on intensity levels and individual psychological resources such as self-efficacy [28]. We therefore examine whether anxiety mediates framing effects while acknowledging its potential to produce both adaptive and maladaptive responses.

Loss-framed messages, which emphasize the adverse consequences of inaction, are especially likely to elicit emotional arousal compared to gain-framed messages that focus on positive outcomes [29]. A meta-analysis of 61 framing studies in environmental communication revealed that loss-framed appeals were generally more effective in shaping intentions and behaviors, particularly through the evocation of negative emotions such as anxiety and fear [30]. CCA, once activated, can serve as a powerful motivator for pro-environmental behavior. Evidence from large-scale cross-national studies has demonstrated a positive relationship between individuals’ climate-related anxiety and their engagement in sustainable behaviors such as energy conservation, ethical consumption, and voluntary carbon offsetting [31]. In many cases, individuals respond to CCA by increasing low-carbon behavioral intention as a way to cope with distress and restore a sense of agency.

Building on this literature, we argue that climate change anxiety may mediate the relationship between message framing and VEI. Specifically, loss-framed messages are more likely than gain-framed ones to trigger CCA. When anxiety levels remain within a functional range and individuals possess adequate coping resources (e.g., self-efficacy), this emotional response may translate into engagement intentions rather than avoidance. Thus, we propose the following hypothesis:

*H2: The loss-framed message will elicit higher levels of CCA than the gain-framed message*.

*H3: CCA will positively influence VEI toward PCT.*

### The moderating role of felt responsibility

Importantly, individuals do not uniformly respond to climate-framed messages or the emotional responses they evoke. One factor that may shape this variability is FR, which reflects an individual’s perceived obligation—whether personal, social, or moral—to take action in response to climate change. People with a strong sense of responsibility tend to view climate threats as directly relevant to themselves, often interpreting environmental issues as ethical imperatives that demand personal engagement [32]. Research has shown that individuals who report higher levels of FR are generally more motivated to engage in climate mitigation behaviors [33]. For instance, promoting a sense of moral duty among students has been linked to increased participation in environmentally responsible actions [34]. When such individuals encounter loss-framed messages, which highlight the risks and consequences of inaction, they may be especially prone to emotional reactions such as anxiety or guilt—since they perceive these consequences as personally meaningful and preventable. In contrast, individuals who do not feel personally responsible for climate issues may process the same messages with psychological distance or disengagement. Therefore, we argue that FR moderates the relationship between message framing and CCA. Specifically, loss-framed messages are more likely to induce anxiety among individuals who feel a stronger sense of personal responsibility for addressing climate change, while the same messages may evoke weaker emotional responses among those low in responsibility.

Furthermore, individuals with high FR often process climate-related messages through a morally engaged lens, perceiving the consequences of inaction as personally relevant and ethically significant [35]. For these individuals, loss-framed messages, which highlight the negative outcomes of not acting (e.g., increased emissions or climate harm), resonate more strongly. Such messages may elicit greater emotional arousal—such as guilt or urgency—thereby increasing motivation to engage in voluntary pro-environmental actions like carbon trading [36]. In contrast, individuals with low FR are less likely to view climate change as their personal duty. As a result, gain-framed messages, which emphasize the potential benefits of action (e.g., contributing to a healthier environment), may be more effective for this group. When intrinsic motivation is low, stronger external incentives are necessary to encourage participation in PCT. From this perspective, the alignment between the message framing and the individual’s internal motivational structure plays a critical role in shaping behavioral responses [37]. Therefore, we propose that FR moderates the relationship between message framing and VEI. Specifically, loss-framed message is expected to be more effective among individuals with high FR, while gain-framed message may be more persuasive among those with lower levels of responsibility. Based on the above discussion, we propose the following hypothesis:

*H4a: FR will moderate the relationship between message framing and VEI. Specifically*,* the loss-framed message will be more effective for individuals with high FR*,* whereas the gain-framed message will be more effective for those with low FR.*

*H4b: FR will moderate the relationship between message framing and CCA. Specifically*,* the effect of the loss-framed message on CCA will be stronger for individuals with high FR than for those with low FR.*

### The moderating role of green self-efficacy

GSE—defined as an individual’s belief in their ability to effectively engage in pro-environmental actions—plays a crucial role in shaping how people respond to climate-related emotions such as anxiety [38], It has been widely recognized as a key psychological driver of sustained environmental behavior [39]. Individuals with high levels of self-efficacy tend to be more motivated, persistent, and confident in their capacity to address environmental issues [40]. Prior studies consistently show that GSE is positively associated with green behaviors, including energy conservation, recycling, and low-carbon lifestyle choices [41]. According to fear appeal theories, individuals must believe that they have the capacity to mitigate a threat in order for fear to lead to constructive action; otherwise, fear may result in psychological disengagement or avoidance [42]. Supporting this view, empirical research has demonstrated that those with high GSE are more likely to respond to CCA with proactive behaviors, while individuals low in efficacy are more prone to helplessness or withdrawal [33]. In other words, self-efficacy serves as a psychological bridge that allows emotional responses like anxiety to be channeled into action. Without such a belief in one’s own ability, anxiety may fail to produce meaningful behavioral outcomes.

In the other way, those with high GSE perceive themselves as capable of making meaningful contributions to environmental protection and are therefore more likely to act upon messages that emphasize the risks of inaction. In particular, loss-framed messages, which highlight the negative consequences of failing to act, tend to evoke stronger behavioral responses among high-efficacy individuals because such messages are perceived as both personally relevant and actionable [43]. Their confidence enables them to interpret climate threats not as paralyzing, but as challenges that they can help resolve—thus transforming concern into proactive engagement [44]. Conversely, individuals with low GSE may view the same loss-framed messages as intimidating or demotivating, since they do not believe their actions will make a significant difference [45]. For these individuals, gain-framed messages, which emphasize the potential benefits of action, may be more persuasive. Research has shown that gain-framed messages tend to be more persuasive for individuals with low levels of self-efficacy [46]. Based on the above discussion, we propose the following hypothesis:

*H5a: GSE will moderate the relationship between message framing and VEI. Specifically*,* the loss-framed message will be more effective for individuals with high GSE*,* whereas the gain-framed message will be more effective for those with low GSE.*

*H5b: GSE will moderate the relationship between CCA and VEI. Specifically*,* the positive effect of CCA on VEI will be stronger for individuals with high GSE than for those with low GSE.*

**Fig. 1:**
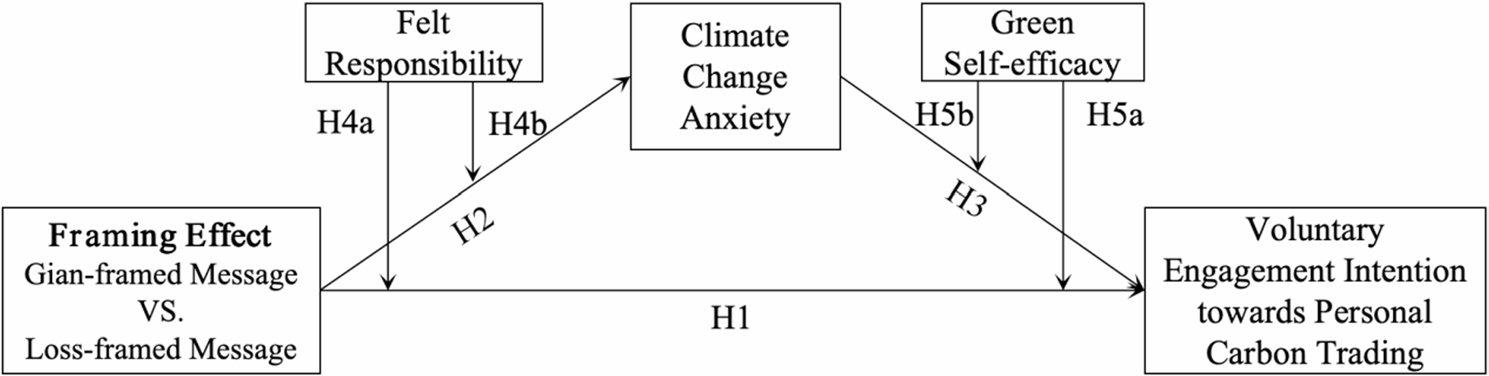
Theoretical model

The constructed conceptual model is illustrated in Fig. 1.

## Methods

### Data collection

An online experiment was conducted in mainland China from April 1 to April 15, 2025. 900 Chinese citizens eligible for PCT were randomly recruited through a leading Chinese online experimental management platform (Wenjuanxing, www.wjx.cn). To ensure sample representativeness, we employed quota sampling to maintain a relatively balanced gender distribution. Participants were informed that this was an academic study on personal carbon trading participation conducted by university researchers. To minimize demand effects, participants were not informed that message framing was the experimental manipulation. To incentivize conscientious participation, participants were required to complete a structured questionnaire and were offered the opportunity to receive modest monetary rewards through a lottery system upon completion of the experimental tasks. All returned questionnaires underwent a rigorous screening process, including attention check verification, IP address uniqueness verification, and a minimum completion time threshold of 150 s. 112 participants either withdrew from the experiment or failed to pass the screening process. The final dataset comprised 788 valid responses, yielding an effective response rate of 87.56%. Notably, our data collection relied entirely on anonymous questionnaires, with no personal identifiers collected. Participants accessed an online survey platform where they were first presented with a comprehensive disclosure statement. This statement outlined the research objectives, methodological approach, associated risks and rewards, privacy protection protocols, and emphasized the voluntary nature of participation.

The demographic characteristics of the participants were as follows: In terms of gender distribution, there were 447 males and 341 females, representing a ratio of 56.73% to 43.27%. The majority of participants (*N* = 566, 71.83%) were aged between 26 and 45 years. Regarding education level, 4.31% of participants held Master’s degrees or above, 66.75% had College or Bachelor’s degrees, and 28.93% had completed high school education or below. With respect to annual income levels, the majority of respondents (*N* = 551, 69.92%) reported annual earnings between 32,196 and 95,055 RMB.

Concerning awareness and experience with PCT: 59.01% of participants indicated no prior knowledge of PCT mechanisms, while 37.56% reported previous experience with PCT-related activities (e.g., participation in carbon inclusion platforms or carbon credit exchange programs). Additionally, 46.57% of participants demonstrated awareness of China’s twin goals of carbon peaking and carbon neutrality. The detailed demographic characteristics of the participants are presented in Table 1.

**Table 1:** Demographic profile of respondents

Items	Category	Frequency (*N* = 788)	Frequency in gain-frame (*N* = 394)	Frequency in loss-frame (*N* = 394)
Gender	Male	447	230	177
	Female	341	164	217
Age	18–25	68	33	35
	26–35	318	156	162
	36–45	248	127	121
	46–55	106	62	44
	56 and above	48	16	32
Education level	High school and below	228	150	78
	College & Bachelor’s degree	526	225	301
	Master’s degree and above	34	19	15
Annual income (CNY)	9215 and below	32	26	6
	9215–20,442	48	22	26
	20,443–32,195	86	46	40
	32,196–50,220	257	108	149
	50,221–95,055	294	154	140
	95,055 and above	71	38	33

### Stimuli

This study employed a two-stage experimental stimulus presentation approach. Initially, all participants read a neutral introductory material about PCT, which objectively described the fundamental concepts and operational mechanisms of PCT to ensure participants had a basic understanding of the program. For instance, If carbon emissions are below the allocated quota, PCT users can sell their remaining carbon credits for profit. The complete introduction is presented in Appendix A. Subsequently, participants were randomly assigned to either a gain-framed group or a loss-framed group, receiving differently framed PCT promotional messages. Both sets of information maintained consistency in structure and complexity, differing only in their framing orientation. The gain-framed group emphasized the environmental benefits of participating in PCT, such as “By participating in PCT, you will directly contribute to reducing CO2 emissions.” Conversely, the loss-framed group emphasized potential environmental losses from non-participation, such as “By foregoing participation in PCT, additional CO2 emissions will be generated annually.” To ensure the effectiveness of the experimental manipulation, we adhered to the following principles in designing the stimulus materials: (1) Information equivalence: Both framing groups contained identical information content and specific data, differing only in presentation style; (2) Structural consistency: Maintaining identical sentence structures and linguistic complexity; (3) Comprehensibility: Considering potential heterogeneity in participants’ comprehension abilities, we employed clear and easily understandable expressions; (4) Exposure time control: The viewing duration for promotional messages was fixed at 30 s for both groups, during which participants could independently read the framing message to ensure thorough comprehension [47]. The complete framing information is presented as followed for the manipulation of the messages [48]:

*Gain-framed message: By participating in PCT*,* you will directly help reduce CO2 emissions. Through your active engagement*,* air quality is expected to improve within years*,* creating a healthier environment for future generations.*

*Loss-framed message: By not participating in PCT*,* additional CO2 emissions will be generated each year. If we don’t act now*,* air quality may deteriorate within years*,* threatening the healthy living environment of future generations.*

By having participants first receive neutral introductory information before the framing treatment, we effectively controlled for confounding effects arising from differences in prior knowledge.

### Procedure and measures

This study employed a randomized group experimental design with a total of 788 participants. Through random assignment, participants were evenly divided into a gain-framed group (*n* = 394) and a loss-framed group (*n* = 394). Participants in the gain-framed group received framed messages emphasizing the positive outcomes of pro-environmental behaviors (i.e., positive contributions to environmental protection), while those in the loss-framed group received framed messages highlighting the consequences of environmental degradation (i.e., negative impacts on the ecological environment). Prior to the formal experiment, all participants first read a neutral descriptive material about PCT, which objectively introduced the basic concepts, operational mechanisms, and participation methods of PCT. We conducted a pre-test using three items regarding Basic Information (BI) to measure participants’ baseline cognition and participation experience regarding PCT, including: (BI1) I am aware of the basic concept of PCT; (BI2) I have participated in PCT related activities; and (BI3) I am aware of China’s twin goals of carbon peaking and carbon neutrality. To verify manipulation effectiveness, participants then rated message framing on a single item: “The message I just read emphasized:” (1 = negative outcomes of not participating, 7 = positive outcomes of participating).

After completing the pre-test questionnaire, participants from different groups were exposed to either gain-framed or loss-framed messages promoting adoption of PCT within a specified time frame. Subsequently, participants completed a structured post-test questionnaire designed to measure four key constructs: CCA, VEI, FR, and GSE. This measurement phase was instrumental in analyzing the mechanism through which message framing influences voluntary participation intention in PCT.

Given that these items were adapted from previously validated English scales with positive reporting outcomes, the research team implemented a rigorous translation-back-translation procedure to ensure cross-cultural validity of the measurement instruments. Specifically, the research team first translated the validated English scales into Chinese and make appropriate adjustments to align with the PCT context. The items were then optimized based on feedback from a pilot sample (*N* = 38). Subsequently, two independent bilingual experts, who were not involved in the initial translation, back-translated the Chinese version into English. The research team evaluated and adjusted the questionnaire by comparing the back-translated version with the original scales, focusing on semantic equivalence, conceptual equivalence, and cultural adaptation, ultimately finalizing the formal questionnaire. The questionnaire required approximately 3–10 min to complete. The measurement instrument comprised 30 items designed to assess the proposed four core constructs. All measurement items were derived from existing literature and underwent meticulous adaptation to ensure their applicability to the PCT research context. All constructs were measured using standardized 7-point Likert scales (1 = strongly disagree, 7 = strongly agree). This unified measurement approach not only enhanced the internal consistency of the scales but also facilitated respondent comprehension and response, while providing a reliable metric foundation for subsequent data analysis. Specifically, five items (FR1-FR5) were employed to measure the FR variable, which were adapted from [32]. For instance, one item states “I believe participating in PCT is important for addressing climate change”. Thirteen items (CCA1-CCA13) were utilized to measure the CCA variable, adapted from [49], with a sample item being “Thinking about climate change makes it difficult for me to concentrate.” The GSE variable was measured using six items (GSE1-GSE6), which were adapted from [50] and [38], exemplified by the statement “I feel I can succeed in accomplishing environmental ideas.” Additionally, six items (VEI1-VEI6) were used to measure the VEI variable, adapted from [13] and [51], with a representative item being “I will often participate in low-carbon activities through the PCT platform”.

Finally, as shown in Table 1, participants were required to provide four Demographic Information (DI) items, including (DI1) gender; (DI2) age group; (DI3) highest education level; and (DI4) annual income range (CNY). Throughout the experimental procedure, we attempted to enhance internal validity by controlling and limiting participants’ discussion of the experiment, and minimize demand characteristics by preventing exposure to other participants.

The specified scale information is summarized in Appendix A.

### Data analysis

This study employs a multi-step analytical approach to examine the theoretical model of individuals’ voluntary participation in PCT. First, to assess the direct impact of message framing on VEI, we conducted Analysis of Covariance (ANCOVA) using Python 3.13 while controlling for relevant covariates. Subsequently, we evaluated the measurement model through Confirmatory Factor Analysis (CFA) using IBM SPSS Statistics 25 to assess the reliability, convergent validity, and discriminant validity of the measurement instruments. Following this, we utilized Hayes’s PROCESS macro (Model 4 & Model 29) in IBM SPSS Statistics 25 to conduct path analysis, mediation analysis, and moderated mediation analysis [52, 53]. Interaction effect plots were generated to visually demonstrate the moderating effects of FR and GSE. Finally, given preliminary diagnostics indicated non-normal residual distribution, we employed PLS-SEM as a robustness check. Unlike PROCESS, which assumes normality, PLS-SEM is distribution-free and suitable for non-normal data with a complex theoretical model incorporating both mediation and moderation effects [54]. Convergent results across methods would indicate findings are robust to distributional assumptions and analytical approach.

## Results

### Results of measurement model assessment

Firstly, manipulation check confirmed successful framing. Gain-framed group participants rated messages as more gain-framed (M = 5.47, SD = 1.23) than loss-framed group (M = 2.81, SD = 1.19), t(786) = 30.42, *p* < 0.001, Cohen’s d = 2.19. This large effect indicates participants correctly perceived framing valence as intended.

The study employed a seven-point Likert scale to measure four constructs in the conceptual model: CCA, VEI, FR, and GSE. Prior to data analysis, we conducted reliability and validity tests to ensure the measurement instrument’s effectiveness. Initially, reliability analysis revealed an overall Cronbach’s α of 0.953 for the questionnaire, with all constructs’ values exceeding 0.7: 0.938 (CCA), 0.913 (VEI), 0.818 (FR), and 0.821 (GSE). These results indicate high reliability and strong internal consistency across all constructs. Subsequently, a CFA was conducted. Before proceeding with the CFA, the Kaiser-Meyer-Olkin (KMO) measure and Bartlett’s test of sphericity were employed to assess the dataset’s suitability for factor analysis. The results yielded a KMO value of 0.971, surpassing the threshold of 0.8, while Bartlett’s test of sphericity demonstrated statistical significance (*p* < 0.001), confirming the dataset’s appropriateness for CFA. The CFA results revealed that, with the exception of item GSE1 (0.653), all items exhibited factor loadings greater than 0.7, ranging from 0.717 (GSE5) to 0.869 (VEI1). While GSE1’s loading falls below the conventional 0.70 threshold, we retained it because the overall GSE construct demonstrated adequate validity (CR = 0.890, AVE = 0.528, α = 0.821), and loadings above 0.60 are considered acceptable when construct-level metrics are strong [54]. Sensitivity analysis indicated removing GSE1 would marginally improve AVE to 0.548 and CR to 0.905, gains insufficient to justify content loss. Furthermore, the composite reliability (CR) scores for all constructs exceeded the 0.7 threshold, specifically: 0.946 (CCA), 0.932 (VEI), 0.873 (FR), and 0.890 (GSE).

As shown in Table 2, the measurement scale properties demonstrate high reliability overall. Subsequently, we assessed the validity of the measurement model. The Average Variance Extracted (AVE) values for all constructs exceeded the recommended threshold of 0.5, specifically 0.576 (CCA), 0.696 (VEI), 0.578 (FR), and 0.528 (GSE), indicating acceptable convergent validity. To evaluate discriminant validity, we employed the Fornell-Larcker criterion [55]. As presented in Table 3, the square root of AVE for each construct (reported on the diagonal of the correlation matrix) was greater than the absolute values of its correlations with other constructs, demonstrating acceptable discriminant validity. Furthermore, we examined multicollinearity among all measured items. The Variance Inflation Factor (VIF) values for all items were below the threshold of 5, ranging from 1.401 (GSE1) to 2.867 (VEI1), suggesting no obvious multicollinearity issues among the measured items.

**Table 2:** Measurement scale properties

Construct	Items	Factor loading	C. A.	CR	AVE
Climate change anxiety (CCA)	CCA1	0.780	0.938	0.946	0.576
	CCA2	0.761			
	CCA3	0.758			
	CCA4	0.742			
	CCA5	0.720			
	CCA6	0.722			
	CCA7	0.794			
	CCA8	0.735			
	CCA9	0.747			
	CCA10	0.784			
	CCA11	0.769			
	CCA12	0.790			
	CCA13	0.757			
Voluntary engagement intention towards personal carbon trading (VEI)	VEI1	0.869	0.913	0.932	0.696
	VEI2	0.839			
	VEI3	0.826			
	VEI4	0.814			
	VEI5	0.828			
	VEI6	0.829			
Felt responsibility (FR)	FR1	0.767	0.818	0.873	0.578
	FR2	0.753			
	FR3	0.740			
	FR4	0.782			
	FR5	0.760			
Green self-efficacy (GSE)	GSE1	0.653	0.821	0.890	0.528
	GSE2	0.748			
	GSE3	0.769			
	GSE4	0.749			
	GSE5	0.717			
	GSE6	0.719			

*C. A.* Cronbach’s alpha, *AVE* average variance extracted, *CR* composite reliability

**Table 3:** Fornell-Larcker criterion results

Constructs	CCA	FR	GSE	VEI
CCA	0.759			
FR	0.671	0.760		
GSE	0.609	0.556	0.727	
VEI	0.678	0.501	0.509	0.834

### Results of ANCOVA

To examine whether message framing significantly influences individuals’ willingness to participate in PCT, this study conducted an ANCOVA with demographic information (DI1-DI4) as covariates. The independent variable, message framing, was coded as a 0–1 variable, with gain-framed group coded as 1 and loss-framed group as 0. DI1-DI4 were all categorical variables. For gender, male was coded as 1 and female as 0; age, education level, and income were categorical variables coded from low to high as 1–5, 1–3, and 1–6 respectively. All categorical variables were set as dummy variables during the analysis. First, the basic model assumptions were tested. The Durbin-Watson value was 2.175, approximating 2, indicating relative independence of residuals. Although both the Omnibus test (*p* < 0.001) and Jarque-Bera test (JB = 83.972, *p* < 0.001) suggested slight deviations from normality in residual distribution, considering the large sample size (*N* = 788), these deviations should not significantly affect the reliability of analytical results according to the Central Limit Theorem [56]. The ANCOVA results revealed a significant effect of message framing on VEI [F(1,782) = 9.723, *p* < 0.001]. Furthermore, none of the demographic variables demonstrated significant effects on the dependent variable [DI1 (β = 0.0021, *p* = 0.981), DI2 (β = −0.0303, *p* = 0.480), DI3 (β = −0.0868, *p* = 0.304), DI4 (β = −0.0246, *p* = 0.501)]. Post-hoc mean comparisons with Bonferroni correction indicated that the gain-framed group exhibited significantly lower behavioral intention (M = 3.99, SE = 0.038) compared to the loss-framed group (M = 4.58, SE = 0.078). This difference was further validated through t-test analysis (t = −6.834, *p* < 0.001). The empirical findings support that message framing has a significant impact on individuals’ willingness to participate in carbon trading, with loss-framed promotional messages effectively enhancing individuals’ participation intention in PCT, thereby supporting H1.

### Results of path & mediation analysis

Hayes’s PROCESS Model 4 [52] was employed to examine the mediating effect of CCA in the relationship between message framing and VEI. All coefficients reported are unstandardized with standard errors. The path analysis results revealed that in the total effect model, message framing exhibited a significant negative direct effect on VEI (β = −0.593, S.E. = 0.087, *p* < 0.001, 95% CI [−0.763, −0.422]), with the model demonstrating statistical significance [F(1, 786) = 46.706, *p* < 0.001]. After incorporating the mediating variable CCA, message framing maintained a significant direct effect on VEI (β = −0.344, S.E. = 0.066, *p* < 0.001, 95% CI [−0.472, −0.215]), further supporting H1 and indicating that the loss-framed group demonstrated higher VEI compared to the gain-framed group. Subsequently, message framing showed a significant negative influence on CCA (β = −0.424, S.E. = 0.098, *p* < 0.001, 95% CI [−0.617, −0.231]), supporting H2 and suggesting that the loss-framed group exhibited higher CCA compared to the gain-framed group. Furthermore, CCA demonstrated a significant positive effect on VEI (β = 0.587, S.E. = 0.024, *p* < 0.001, 95% CI [0.5408, 0.6332]), supporting H3 and indicating that higher levels of CCA were associated with higher levels of VEI. The overall model demonstrated statistical significance [F(2, 785) = 352.8907, *p* < 0.001] and explained 47.34% of the variance in the dependent variable (R² = 0.473). The path analysis results suggest that CCA partially mediates the relationship between message framing and VEI.

Therefore, we further conducted a mediation analysis using the bootstrapping method (with 5,000 sub-samples). The results revealed a significant mediating effect of CCA (Effect = −0.249, BootSE = 0.054, 95% CI [−0.352, −0.141]). The path coefficients indicated that message framing influenced VEI through both direct (β = −0.275) and indirect (β = −0.199) pathways. Furthermore, after including DI1-DI4 as covariates, all path relationships and mediation effects remained significant, demonstrating the robustness of our findings. The path and mediation analyses suggested that CCA partially mediated the relationship between message framing and VEI. Specifically, loss-framed group (compared to gain-framed group) not only directly enhanced VEI but also indirectly promoted VEI by increasing individuals’ CCA.

### Results of mediated moderation analysis

Hayes’s PROCESS Model 29 [52] was employed to examine the conditional mechanism through which message framing influences PCT adoption intention via CCA, with FR as the moderator. Note that PROCESS reports unstandardized coefficients (denoted as β in Hayes’s notation) with standard errors and 95% confidence intervals. The analysis revealed that both message framing (β = −0.9864, SE = 0.2320, t = −4.253, *p* < 0.001) and FR (β = 0.621, SE = 0.042, t = 14.877, *p* < 0.001) significantly predicted CCA. More importantly, a significant interaction effect between message framing and FR was observed (β = 0.152, SE = 0.055, t = 2.743, *p* < 0.01). Simple slope analysis demonstrated that the negative effect of message framing on CCA was strongest at low levels of FR (M-1SD; Effect = −0.652, *p* < 0.001) and weakest at high levels of FR (M + 1SD; Effect = −0.196, *p* < 0.05). The overall model accounted for 47.31% of the variance in CCA [R² = 0.473, F(3, 784) = 234.623, *p* < 0.001]. These findings provide support for H4b.

When VEI serves as the dependent variable, with message framing as the independent variable, CCA as the mediating variable, and FR and GSE as moderating variables, the results revealed more complex moderation patterns. message framing (β = 2.853, SE = 0.201, t = 14.191, *p* < 0.001), CCA (β = 0.571, SE = 0.077, t = 7.464, *p* < 0.001), FR (β = 0.212, SE = 0.039, t = 5.384, *p* < 0.001), and GSE (β = 0.559, SE = 0.075, t = 7.449, *p* < 0.001) all demonstrated significant effects on VEI. The moderation analysis indicated that the interaction effects between message framing and FR (β = −0.246, SE = 0.053, t = −4.624, *p* < 0.001), as well as between message framing and GSE (β = −0.543, SE = 0.052, t = −10.543, *p* < 0.001), significantly influenced VEI. Therefore, H4a and H5a were empirically supported. Additionally, the interaction effect between CCA and GSE did not reach statistical significance (β = −0.033, SE = 0.017, t = −1.921, *p* = 0.055, 95% CI [−0.0670, 0.0007]). Simple slope analysis demonstrated that the positive effect of CCA on VEI remained statistically significant across different levels of GSE, including under low GSE (M-1SD) conditions (Effect = 0.493, SE = 0.043, *p* < 0.001, 95% CI [0.4099, 0.5767]), moderate GSE conditions (Effect = 0.416, SE = 0.032, *p* < 0.001, 95% CI [0.3539, 0.4782]), and high GSE (M + 1SD) conditions (Effect = 0.388, SE = 0.039, *p* < 0.001, 95% CI [0.3123, 0.4645]). This indicates that the impact of CCA on VEI does not significantly vary across different individual GSE levels, thus H5b was not supported. Conditional effects analysis further revealed that the direct effect of message framing was jointly moderated by FR and GSE. Specifically, under conditions of low FR (M-1SD) and low GSE (M-1SD), message framing exhibited a significant positive effect (Effect = 1.0435, *p* < 0.001); whereas under conditions of high FR (M + 1SD) and high GSE (M + 1SD), this effect transformed into a significant negative relationship (Effect = −1.416, *p* < 0.001). The model explained 64.21% of the variance in the dependent variable [R² = 0.642, F(7, 780) = 199.913, *p* < 0.001].

**Fig. 2:**
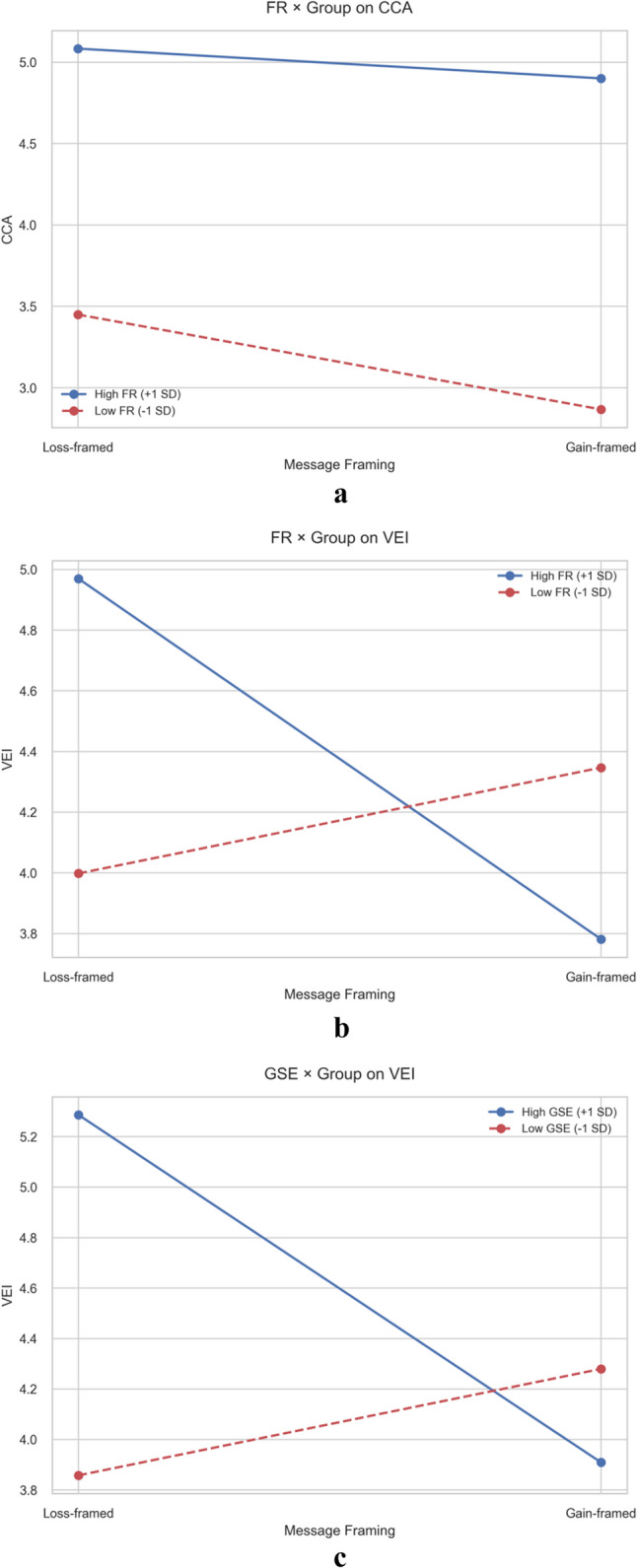
**a** Two-way interaction plot of FR × Group on CCA. **b** Two-way interaction plot of FR × Group on VEI. **c** Two-way interaction plot of GSE × Group on VEI

To aid interpretation, we plotted interaction effects (Fig. 2a and c). In each figure, the x-axis represents message framing conditions (0 = loss frame, 1 = gain frame), the y-axis shows the outcome variable, and separate lines represent high (M + 1SD) versus low (M-1SD) moderator levels. Moderation is indicated by non-parallel lines: diverging or crossing lines signal that framing effects differ across moderator levels.

Figure 2a illustrates how FR moderates framing effects on CCA. Two key patterns emerge. First, both lines slope downward, indicating CCA decreases when moving from loss to gain-framed messages regardless of FR level. However, the low FR line (dashed) shows a steeper decline than the high FR line (solid), demonstrating stronger framing sensitivity among low-FR individuals. Second, the high FR line consistently sits above the low FR line, meaning high-FR individuals experience greater climate anxiety overall. Substantively, this suggests individuals with strong environmental responsibility maintain elevated anxiety even under gain framing, while low-FR individuals’ anxiety is more malleable by message tone. Figure 2b depicts how FR moderates framing effects on VEI, revealing a crossover interaction. For high-FR individuals (solid line), VEI decreases sharply from loss to gain-framed messages—loss-framed messages are substantially more motivating. For low-FR individuals (dashed line), the pattern reverses slightly: VEI increases modestly from loss to gain-framed messages, suggesting gain-framed messages may be marginally more effective. The crossing pattern indicates optimal framing depends on audience responsibility levels: loss-framed messages excel for high-FR groups, while gain-framed messages show relative advantages for low-FR groups. Figure 2c shows GSE’s moderating role on framing-VEI relationships, displaying a similar crossover pattern. High-GSE individuals (solid line) exhibit substantially higher VEI under loss-framed messages than gain-framed messages—the line slopes steeply downward. Low-GSE individuals (dashed line) show minimal framing sensitivity, with slightly higher VEI under gain-framed messages. This pattern suggests that individuals confident in their environmental capabilities respond more strongly to threat-based messaging, whereas those lacking confidence benefit more from opportunity-focused appeals. Practically, this underscores the importance of matching message tone to audience self-efficacy profiles.

### Results of PLS-SEM

Given the non-normal distribution of the questionnaire data, we employed PLS-SEM, which is particularly suitable for handling non-normally distributed data, to construct an identical theoretical model and further examine the PROCESS results [57]. The model fit evaluation revealed that the Standardized Root Mean Square Residual (SRMR) was 0.092, which is below the threshold of 0.1; the geodesic distance (d_G) was 0.554, falling below 0.95; and the Normed Fit Index (NFI) was 0.898, exceeding 0.8. These indices collectively suggest acceptable model fit [58]. While NFI was marginally below 0.90, the convergence of other indices supports acceptable overall model fit. All path coefficients reported from PLS-SEM are standardized.

**Fig. 3:**
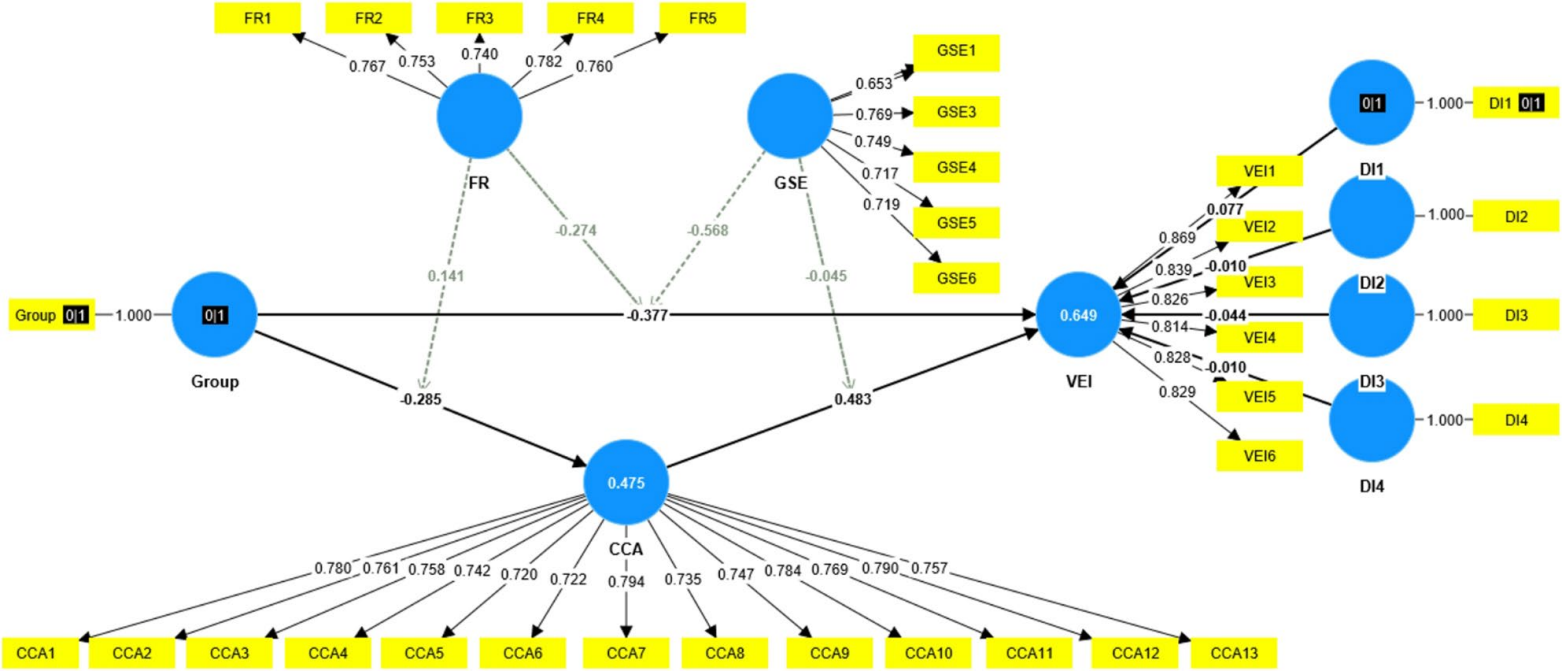
Graphic output of PLS-SEM

Path analysis results (See Fig. 3; Table 4) revealed that message framing exhibited a significant direct negative effect on VEI (β = −0.377, *p* < 0.001), as well as an indirect effect through CCA: message framing significantly and negatively influenced CCA (β = −0.285, *p* < 0.001), while CCA demonstrated a significant positive effect on VEI (β = 0.483, *p* < 0.001). Furthermore, both FR and GSE significantly moderated the influence paths of message framing: FR moderated not only the effect of message framing on VEI (β = −0.274, *p* < 0.01) but also its effect on CCA (β = 0.141, *p* < 0.01); GSE significantly moderated the relationship between message framing and VEI (β = −0.568, *p* < 0.001), whereas its interaction effect with CCA did not reach statistical significance (β = −0.045, *p* > 0.05). These findings lend support to hypotheses H1-H5a, while H5b was not supported, indicating that message framing influences VEI through both direct and indirect pathways, with this influence being moderated by FR and GSE. Furthermore, the mediating effect of CCA was found to be significant (effect = −0.138, SD = 0.028, T = 4.941, *p* < 0.001), with 47.5% and 64.9% of the variance explained in CCA and VEI respectively. In summary, the relationships among all variables demonstrated high consistency across both PLS-SEM and PROCESS methodologies, suggesting robust research findings.

**Table 4:** Direct path analysis

Pathes	Path coefficient	Standard deviation	T statistics	Support
H1: Group ->VEI	−0.377***	0.054	7.018	Yes
H2: Group ->CCA	−0.285***	0.052	5.499	Yes
H3: CCA ->VEI	0.483***	0.04	12.195	Yes
H4a: FR × Group ->VEI	−0.274***	0.07	3.88	Yes
H4b: FR × Group ->CCA	0.141**	0.051	2.734	Yes
H5a: GSE × Group ->VEI	−0.568***	0.064	8.852	Yes
H5b: GSE × CCA->VEI	−0.045	0.029	1.57	No

*p < 0.05; **p < 0.01; ***p < 0.001

## Discussion

This study examined the psychological mechanisms through which message framing influences intentions to participate in personal carbon trading, with particular attention to the mediating role of climate anxiety and the moderating functions of felt responsibility and green self-efficacy. Results from 788 participants demonstrate that loss-framed messages outperform gain-framed messages (M_loss_ = 4.58 vs. M_gain_ = 3.99, *p* < 0.001), with climate anxiety partially mediating this relationship. Importantly, framing effectiveness varies by psychological trait: loss-framed messages prove more effective for high FR/GSE individuals, while gain-framed messages show advantages for low FR/GSE groups. These patterns were consistent across PROCESS and PLS-SEM analyses.

### Theoretical discussion

While PCT participation constitutes pro-environmental behavior, this study addresses context-specific theoretical gaps in market-based environmental instruments. By drawing on Prospect Theory, Norm Activation Theory, and Self-Efficacy Theory, this study demonstrates how established psychological frameworks operate differently in complex policy contexts, contributing to theoretical refinement rather than proposing entirely new mechanisms.

First, the study validates the superiority of loss-framed over gain-framed condition in stimulating VEI (M_loss_ = 4.58 vs. M_gain_ = 3.99), which aligns with the core proposition of Prospect Theory that individuals are more sensitive to losses than equivalent gains [59]. Loss-framed message more effectively motivates individual action by triggering loss aversion psychology regarding avoiding environmental deterioration [60]. This finding extends the application of message framing in low-carbon behavior domains, demonstrating its applicability in complex policy instruments such as PCT. Furthermore, the partial mediating effect of CCA (β = −0.249) reveals the significance of emotional mechanisms in message framing. This result resonates with Eco-Anxiety theory, suggesting that environmental threat information may drive behavioral responses through emotional pathways such as anxiety [61, 62]. However, our positive findings likely reflect moderate anxiety levels induced by our experimental stimuli and the presence of efficacy-enhancing factors in our context. This underscores that anxiety’s motivational function is conditional rather than universal.

In contrast to merely emphasizing the negative effects of anxiety, this study further demonstrates that CCA can be transformed into a driving force for low-carbon behavioral intention under specific conditions (such as loss-framed conditions), providing empirical support for the concept of “adaptive anxiety” [63]. Moreover, the moderating effects of FR and GSE deepen our understanding of individual heterogeneity. FR significantly moderates the impact of message framing on CCA and VEI, which aligns with the mechanism of “responsibility attribution driving pro-environmental behavioral intention” in norm activation theory [64]. Meanwhile, GSE’s enhancement of the positive effect of loss-framed message on VEI corresponds with the core hypothesis of self-efficacy theory, suggesting that individuals with high self-efficacy are more inclined to accept challenging information and take action [65]. These findings indicate that the effectiveness of message framing is highly dependent on individual psychological traits, providing theoretical foundation for “personalized information design” [66]. In conclusion, our contribution extends beyond validating established theories in carbon governance contexts. We demonstrate that: (1) loss aversion operates even when economic incentives are present, (2) climate anxiety functions as a constructive motivator rather than merely a negative state, and (3) communication effectiveness is contingent on recipient psychological traits. These findings advance understanding of how affective and cognitive pathways jointly shape behavioral intention in market-based environmental policies.

### Practical discussion

Our findings offer four practical implications for promoting PCT participation intentions. First, optimize message framing design. Loss-framed messages emphasizing environmental consequences of inaction (e.g., air pollution intensification, extreme weather events) more effectively enhance participation intentions than gain-framed messages highlighting economic returns [67]. Policymakers should combine loss-framed messages with specific action prompts (e.g., “Register your carbon account now”) to facilitate behavioral conversion. Second, implement audience segmentation strategies. For high FR/GSE individuals, loss-framed messages align with their risk-averse orientation and action confidence. For low FR/GSE groups, gain-framed messages paired with efficacy-enhancing information (e.g., “75% of users complete carbon trading through simple steps”) prove more effective. Digital platforms can enable targeted delivery based on user behavioral profiles. Third, balance anxiety and efficacy. While loss-framed messages effectively trigger climate anxiety, excessive threat emphasis may cause disengagement [68]. Communication should embed solution-oriented content: pairing emissions consequences with clear participation pathways and success stories. This threat-efficacy balance approach transforms anxiety into actionable motivation [69]. Fourth, leverage technological enablement. Digital platforms (e.g., WeChat carbon mini-programs) can deliver adaptive messaging based on real-time user data. Emerging technologies such as virtual reality simulating climate impacts (e.g., glacier melting scenarios) could enhance loss-framed message emotional impact [70]. Algorithm-driven personalization enables matching framing strategies to individual psychological characteristics.

However, given we measured intentions rather than actual behaviors, field studies validating these strategies’ effectiveness on real participation are essential before large-scale implementation. These evidence-based recommendations provide strategic guidance for promoting voluntary engagement in carbon governance systems.

### Limitations and future research directions

Although this study provides important insights into the application of message framing in PCT, several limitations point to directions for future research. First, our sample exhibits several limitations that warrant consideration. The study exclusively recruited participants from mainland China (*N* = 788), which constrains the generalizability of our findings across different cultural and socioeconomic contexts. Given that environmental attitudes and policy acceptance are culturally embedded, replication studies in Western cultural systems and diverse carbon policy environments are needed to establish the cross-cultural robustness of message framing effects. Furthermore, our sampling approach may have underrepresented rural populations and low-income groups. Future research should purposefully include these demographics to examine whether economic constraints fundamentally alter how individuals respond to gain/loss-frame messages.

First, our sample demonstrates three interrelated limitations that affect generalizability. First, the demographic composition skews toward middle-aged (71.83% aged 26–45) and college-educated individuals (66.75%), likely overrepresenting populations with higher environmental awareness and digital literacy. Second, by recruiting exclusively from mainland China, we cannot determine whether our findings extend to other cultural contexts. Third, our reliance on online recruitment platforms may have systematically excluded rural residents, elderly populations, and economically disadvantaged groups who have limited internet access or lower digital engagement.

These limitations have theoretical implications. Cultural values may shape our findings—Chinese collectivism may amplify felt responsibility effects compared to individualistic cultures where personal obligation plays a weaker role [71]. Political context matters: China’s centralized climate governance and official carbon neutrality commitments may enhance message credibility compared to contexts with contested climate politics or policy skepticism [72]. Urban-rural differences are critical: our likely urban-dominant sample possesses greater market economy exposure and familiarity with digital carbon platforms (e.g., Alipay), potentially reducing cognitive barriers and inflating self-efficacy effects. Rural populations with limited digital access may respond differently to PCT communication. Furthermore, our educational skew raises concerns about cognitive processing. Loss framing effectiveness may diminish among lower-literacy populations who struggle with abstract policy concepts, while anxiety-inducing messages could backfire without adequate informational resources. Future research should employ stratified sampling across education levels, geographic regions (urban/rural), and cultural contexts (collectivist vs. individualistic societies) to establish boundary conditions. Cross-national replication in contexts with different political systems and climate policy environments is essential before generalizing communication strategies.

Second, concerning the short-term nature of experimental design and behavioral gap. This study measured immediate VEI through online experiments but did not track actual behaviors (such as carbon trading frequency or emission reduction). In the environmental protection field, there may be significant discrepancies between behavioral intentions and actual behaviors [73]. Future research could adopt longitudinal designs, incorporating real carbon account data (such as Alipay’s Ant Forest), to analyze the dynamic effects of framing information on long-term behavior. Third, the breadth of mediating and moderating variables. Although the mediating role of CCA has been revealed, other broader potential mechanisms (such as environmental identity or moral obligation) have not been encompassed [74]. An important caveat concerns anxiety measurement and potential threshold effects. Although we measured climate change anxiety as a continuous variable, existing literature suggests that anxiety may exhibit an inverted-U relationship with behavioral outcomes [28]. Our experimental manipulation likely induced moderate anxiety levels, which may explain why we observed a positive mediating effect rather than the debilitating effects associated with extreme anxiety. Future research should examine curvilinear relationships and identify optimal anxiety ranges for motivating intentions without triggering avoidance. Additionally, measuring coping strategies (problem-focused vs. emotion-focused) could clarify when anxiety promotes versus inhibits engagement. Meanwhile, the moderating variables focus on FR and GSE, while individual values (such as collectivistic orientation) or social norms (such as peer pressure) may further moderate the message framing effects [75]. Future research could explore more boundary conditions in the process of how message framing influences individual pro-environmental behaviors.

Fourth, implementation feasibility of practical recommendations. While our findings support tailoring messages to psychological traits (FR and GSE), we acknowledge that measuring these traits at population scale poses practical challenges. Our study demonstrates moderation effects under controlled experimental conditions but does not validate implementation strategies. Future research should examine whether behavioral proxies (e.g., prior carbon platform engagement, environmental action history) can effectively substitute for direct trait measurement in real-world campaigns. Additionally, cost-effectiveness analyses comparing segmented versus uniform messaging strategies are needed to determine scalability of personalized communication approaches.

Finally, our reliance on static textual stimuli leaves unexamined whether alternative communication formats might enhance framing effectiveness. While our design prioritized internal validity and experimental control, multimedia formats (e.g., videos, interactive interfaces) or emerging immersive technologies merit investigation in future research. While speculative and not directly supported by our data, several avenues merit future investigation: (1) immersive technologies (e.g., VR climate impact simulations) may amplify loss-framed message emotional resonance; (2) dynamic personalized framing adjusting content based on real-time user behavior might enhance intervention effectiveness [76]; (3) machine learning algorithms could enable adaptive delivery systems matching message framing to individual psychological characteristics [77]. However, these technological applications require empirical validation examining whether added complexity yields proportional improvements in actual participation behaviors compared to simpler text-based interventions. Such research would inform cost-benefit decisions for large-scale policy implementation.

## Conclusion

This study demonstrates that loss-framed messages more effectively enhance PCT participation intentions than gain-framed messages, validating Prospect Theory in carbon governance contexts. Climate anxiety partially mediates this effect, revealing emotional pathways in climate communication. Importantly, effectiveness varies by psychological trait: loss-framed messages excel for high FR/GSE individuals, while gain-framed messages prove more suitable for low FR/GSE groups. These findings underscore that effective climate communication requires audience segmentation rather than uniform messaging—a critical insight for designing behavior change interventions in voluntary carbon markets.

This research contributes to theory by identifying and validating a framing-anxiety-intention pathway that is contingent on individual psychological characteristics, specifically in the context of market-based environmental policies. From a practical standpoint, our findings support the strategic use of loss-framed messages while emphasizing the importance of audience segmentation based on felt responsibility and green self-efficacy to optimize communication effectiveness. Limitations include sample homogeneity, intention-behavior gap, and single-country context. Future research should validate effects cross-culturally and longitudinally track actual participation behaviors. This study contributes evidence-based communication strategies for promoting voluntary engagement in carbon governance systems.

## Supplementary Information

Supplementary Material 1.

## Data Availability

The datasets used and/or analysed during the current study are available from the corresponding author on reasonable request.

## Abbreviations

PCT: Personal Carbon Trading
CCA: Climate Change Anxiety
FR: Felt Responsibility
GSE: Green SelfEfficacy
VEI: Voluntary Engagement Intention
IPCC: Intergovernmental Panel On Climate Change
BI: Basic Information
DI: Demographic Information
ANCOVA: Analysis Of Covariance
CFA: Confirmatory Factor Analysis
PLS-SEM: Partial Least Squares Structural Equation Modeling
KMO: KaiserMeyerOlkin
C.A.: Cronbachs Alpha
AVE: Average Variance Extracted
CR: Composite Reliability

## Footnotes

1
https://www.ipcc.ch/2022/04/04/ipcc-ar6-wgiii-pressrelease/.

## References

[1] Li C, Chen X, Yuan C. Does digital government reduce carbon emissions? Empirical evidence from global sources. J Environ Manage. 2025;380:125081. 40132373 10.1016/j.jenvman.2025.125081

[2] Chen N, Zhao Y, He H, Ma X, Xu X, Li L, Gang S, Xue B. Stability analysis of carbon emission trading mechanism in China based on a tripartite evolutionary game. Sci Rep. 2025;15(1):7304. 40025130 10.1038/s41598-025-91373-6 PMC11873314

[3] Long Y, Yoshida Y, Jiang Y, Huang L, Wang W, Mi Z, Shigetomi Y, Kanemoto K. Japanese urban household carbon footprints during early-stage COVID-19 pandemic were consistent with those over the past decade. Npj Urban Sustain. 2023;3(1):19. 37009569 10.1038/s42949-023-00095-z PMC10052282

[4] Kenny T, Gray N. Comparative performance of six carbon footprint models for use in Ireland. Environ Impact Assess Rev. 2009;29(1):1–6.

[5] Du M, Zhang X, Xia L, Cao L, Zhang Z, Zhang L, et al. The China carbon watch (CCW) system: a rapid accounting of household carbon emissions in China at the provincial level. Renew Sustain Energy Rev. 2022;155:111825.

[6] Tian J, Sun M, Gong Y, Chen X, Sun Y. Chinese residents’ attitudes toward consumption-side climate policy: the role of climate change perception and environmental topic involvement. Resour Conserv Recycl. 2022;182:106294.

[7] Fan J, Wang S, Wu Y, Li J, Zhao D. Buffer effect and price effect of a personal carbon trading scheme. Energy. 2015;82:601–10.

[8] Bristow AL, Wardman M, Zanni AM, Chintakayala PK. Public acceptability of personal carbon trading and carbon tax. Ecol Econ. 2010;69(9):1824–37.

[9] Frey BS, Oberholzer-Gee F. The cost of price incentives: an empirical analysis of motivation crowding-out. Am Econ Rev. 1997;87(4):746–55.

[10] Capstick, SB, Lewis, A. Effects ofpersonal carbon allowances on decisionmaking: evidence from an experimental simulation. Clim.Policy. 2010;10:369–84.

[11] Wadud Z, Chintakayala PK. Personal carbon trading: trade-off and complementarity between in-home and transport related emissions reduction. Ecol Econ. 2019;156:397–408.

[12] Brekke KA, Kverndokk S, Nyborg K. An economic model of moral motivation. J Public Econ. 2003;87(9–10):1967–83.

[13] Tan X, Wang X, Zaidi SHA. What drives public willingness to participate in the voluntary personal carbon-trading scheme? A case study of Guangzhou Pilot, China. Ecol Econ. 2019;165:106389.

[14] Gao L, Jiang J, He H, Zhou Q, Wang S, Li J. Uncertainty or trust? Political trust, perceived uncertainty and public acceptance of personal carbon trading policy. Environ Geochem Health. 2022;44(9):3157–71. 35129706 10.1007/s10653-022-01214-y

[15] Dai J, Gong S. Sustainable messaging strategies and consumer food waste: the congruence effect between message framing and state anxiety. J Retail Consum Serv. 2024;79:103817.

[16] Mollen S, Holland RW, Ruiter RA, Rimal RN, Kok G. When the frame fits the social picture: the effects of framed social norm messages on healthy and unhealthy food consumption. Commun Res. 2021;48(3):346–78.

[17] Yang D, Lu Y, Zhu W, Su C. Going green: how different advertising appeals impact green consumption behavior. J Bus Res. 2015;68(12):2663–75.

[18] Kai-Ineman D, Tversky A. Prospect theory: an analysis of decision under risk. Econometrica. 1979;47(2):363–91.

[19] Amatulli C, De Angelis M, Peluso AM, Soscia I, Guido G. The effect of negative message framing on green consumption: an investigation of the role of shame. J Bus Ethics. 2019;157(4):1111–32.

[20] Grazzini L, Rodrigo P, Aiello G, Viglia G. Loss or gain? The role of message framing in hotel guests’ recycling behaviour. J Sustainable Tourism. 2018;26(11):1944–66.

[21] Gonzales MH, Aronson E, Costanzo MA. Using social cognition and persuasion to promote energy conservation: a quasi-experiment 1. J Appl Soc Psychol. 1988;18(12):1049–66.

[22] Nabi RL, Gustafson A, Jensen R. Framing climate change: exploring the role of emotion in generating advocacy behavior. Sci Communication. 2018;40(4):442–68.

[23] Zhang M, Zhang G-y, Gursoy D. Fu X-r: message framing and regulatory focus effects on destination image formation. Tour Manag. 2018;69:397–407.

[24] Clayton S, Karazsia BT. Development and validation of a measure of climate change anxiety. J Environ Psychol. 2020;69:101434.

[25] Crandon TJ, Scott JG, Charlson FJ, Thomas HJ. A social–ecological perspective on climate anxiety in children and adolescents. Nat Clim Change. 2022;12(2):123–31.

[26] Hogg TL, Stanley SK, O’Brien LV, Wilson MS, Watsford CR. The Hogg Eco-Anxiety scale: development and validation of a multidimensional scale. Glob Environ Change. 2021;71:102391.

[27] Whitmarsh L, Player L, Jiongco A, James M, Williams M, Marks E, et al. Climate anxiety: what predicts it and how is it related to climate action? J Environ Psychol. 2022;83:101866.

[28] Witte K. Putting the fear back into fear appeals: the extended parallel process model. Commun Monogr. 1992;59(4):329–49.

[29] Bilandzic H, Kalch A, Soentgen J. Effects of goal framing and emotions on perceived threat and willingness to sacrifice for climate change. Sci Commun. 2017;39(4):466–91.

[30] Homar AR, Cvelbar LK. The effects of framing on environmental decisions: a systematic literature review. Ecol Econ. 2021;183:106950.

[31] Ogunbode CA, Doran R, Hanss D, Ojala M, Salmela-Aro K, van den Broek KL, et al. Climate anxiety, wellbeing and pro-environmental action: correlates of negative emotional responses to climate change in 32 countries. J Environ Psychol. 2022;84:101887.

[32] Punzo G, Panarello D, Pagliuca MM, Castellano R, Aprile MC. Assessing the role of perceived values and felt responsibility on pro-environmental behaviours: a comparison across four EU countries. Environ Sci Policy. 2019;101:311–22.

[33] Qin Z, Wu Q, Bi C, Deng Y, Hu Q. The relationship between climate change anxiety and pro-environmental behavior in adolescents: the mediating role of future self-continuity and the moderating role of green self-efficacy. BMC Psychol. 2024;12(1):241. 38678287 10.1186/s40359-024-01746-1 PMC11056057

[34] Dasi AA, Miarsyah M, Rusdi R. The relationship between personal responsibility and pro-environmental intention in high schools’ students. JPBI (Jurnal Pendidikan Biologi Indonesia). 2019;5(1):17–22.

[35] Gifford R. The dragons of inaction: psychological barriers that limit climate change mitigation and adaptation. Am Psychol. 2011;66(4):290. 21553954 10.1037/a0023566

[36] Spence A, Pidgeon N. Framing and communicating climate change: the effects of distance and outcome frame manipulations. Glob Environ Change. 2010;20(4):656–67.

[37] Trope Y, Liberman N. Construal-level theory of psychological distance. Psychol Rev. 2010;117(2):440. 20438233 10.1037/a0018963 PMC3152826

[38] Guo L, Xu Y, Liu G, Wang T, Du C. Understanding firm performance on green sustainable practices through managers’ ascribed responsibility and waste management: green self-efficacy as moderator. Sustainability. 2019;11(18):4976.

[39] Mo Z, Liu MT, Wu P. Shaping employee green behavior: a multilevel approach with Pygmalion effect. Asia Pac J Mark Logistics. 2022;34(2):322–49.

[40] Savari M, Naghibeiranvand F, Asadi Z. Modeling environmentally responsible behaviors among rural women in the forested regions in Iran. Glob Ecol Conserv. 2022;35:e02102.

[41] Khan AN. Elucidating the effects of environmental consciousness and environmental attitude on green travel behavior: moderating role of green self-efficacy. Sustain Dev. 2024;32(3):2223–32.

[42] Armbruster ST, Manchanda RV, Vo N. When are loss-framed messages more effective in climate change communication? An application of fear appeal theory. Sustainability. 2022;14(12):7411.

[43] Florence ES, Fleischman D, Mulcahy R, Wynder M. Message framing effects on sustainable consumer behaviour: a systematic review and future research directions for social marketing. J Social Mark. 2022;12(4):623–52.

[44] Van Zomeren M, Spears R, Leach CW. Experimental evidence for a dual pathway model analysis of coping with the climate crisis. J Environ Psychol. 2010;30(4):339–46.

[45] Witte K, Allen M. A meta-analysis of fear appeals: implications for effective public health campaigns. Health Educ Behav. 2000;27(5):591–615. 11009129 10.1177/109019810002700506

[46] Carfora V, Morandi M, Catellani P. The effect of message framing in promoting the mediterranean diet: the moderating role of eating self-efficacy. Foods. 2022;11(10):1454. 35627024 10.3390/foods11101454 PMC9140873

[47] Rothman AJ, Salovey P. Shaping perceptions to motivate healthy behavior: the role of message framing. Psychol Bull. 1997;121(1):3. 9000890 10.1037/0033-2909.121.1.3

[48] Lim JS, Noh G-Y. Effects of gain-versus loss-framed performance feedback on the use of fitness apps: mediating role of exercise self-efficacy and outcome expectations of exercise. Comput Hum Behav. 2017;77:249–57.

[49] Chan H-W, Tam K-P, Clayton S. Testing an integrated model of climate change anxiety. J Environ Psychol. 2024;97:102368.

[50] Mughal MF, Cai SL, Faraz NA, Ahmed F. Environmentally specific servant leadership and employees’ pro-environmental behavior: mediating role of green self efficacy. Psychol Res Behav Manag. 2022. 10.2147/PRBM.S328776. 35210879 10.2147/PRBM.S328776 PMC8856745

[51] Matekele CK, Kileo NY, Muna AM. Determinants of local communities’ intentions to adopt carbon trading: evidence from Tanzania. Cogent Business & Management. 2024;11(1):2398192.

[52] Hayes, AF. PROCESS: A versatile computational tool for observed variable mediation, moderation, and conditional process modeling. In: University of Kansas, KS. 2012.

[53] Hayes AF. Partial, conditional, and moderated moderated mediation: quantification, inference, and interpretation. Commun Monogr. 2018;85(1):4–40.

[54] Hair JF, Risher JJ, Sarstedt M, Ringle CM. When to use and how to report the results of PLS-SEM. Eur Bus Rev. 2019;31(1):2–24.

[55] Fornell C, Larcker DF. Evaluating structural equation models with unobservable variables and measurement error. J Mark Res. 1981;18(1):39–50.

[56] Kwak SG, Kim JH. Central limit theorem: the cornerstone of modern statistics. Korean J Anesthesiol. 2017;70(2):144. 28367284 10.4097/kjae.2017.70.2.144 PMC5370305

[57] Hair, JF, Hult, GTM., Ringle, CM, Sarstedt, MA. Primer on Partial Least Squares Structural Equation Modeling (PLS-SEM). Publications, SAGE. 2017.

[58] Hu Lt, Bentler PM. Cutoff criteria for fit indexes in covariance structure analysis: conventional criteria versus new alternatives. Struct Equation Modeling: Multidisciplinary J. 1999;6(1):1–55.

[59] Kahneman D, Tversky A. Prospect theory: An analysis of decisionunder risk. Econometrica. 1979;47(2):363-91.

[60] Tversky A, Kahneman D. Loss aversion in riskless choice: a reference-dependent model. Q J Econ. 1991;106(4):1039–61.

[61] Hayes K, Blashki G, Wiseman J, Burke S, Reifels L. Climate change and mental health: risks, impacts and priority actions. Int J Ment Health Syst. 2018;12:1–12. 29881451 10.1186/s13033-018-0210-6 PMC5984805

[62] Lawrance EL, Thompson R, Le Newberry J, Page L, Jennings N. The impact of climate change on mental health and emotional wellbeing: a narrative review of current evidence, and its implications. Int Rev Psychiatry. 2022;34(5):443–98. 36165756 10.1080/09540261.2022.2128725

[63] Gutiérrez-García AG, Contreras CM. Anxiety: an adaptive emotion. New Insights into Anxiety Disorders. 2013;1(2):21–37.

[64] Schwartz SH. Normative influences onaltruism. Advances in experimental social psychology. 1997;10:221-79.

[65] Bandura A. Self-efficacy: toward a unifying theory of behavioral change. Psychol Rev. 1977;84(2):191. 847061 10.1037//0033-295x.84.2.191

[66] Chen M-F, Tung P-J. Developing an extended theory of planned behavior model to predict consumers’ intention to visit green hotels. Int J Hosp Manage. 2014;36:221–30.

[67] Bolderdijk JW, Steg L, Geller ES, Lehman PK, Postmes T. Comparing the effectiveness of monetary versus moral motives in environmental campaigning. Nat Clim Change. 2013;3(4):413–6.

[68] Ojala M. Regulating worry, promoting hope: how do children, adolescents, and young adults cope with climate change? Int J Environ Sci Educ. 2012;7(4):537–61.

[69] Marlon, JR, Bloodhart, B, Ballew, MT, Rolfe-Redding, J, Roser-Renouf, C, Leiserowitz, A, Maibach, E. How hope and doubt affect climate change mobilization. Frontiers in Communication. 2019;4:20.

[70] Ahn SJ, Bostick J, Ogle E, Nowak KL, McGillicuddy KT, Bailenson JN. Experiencing nature: embodying animals in immersive virtual environments increases inclusion of nature in self and involvement with nature. J Comput-Mediat Commun. 2016;21(6):399–419.

[71] Hofstede G. Culture’s consequences: Comparing values, behaviors, institutions and organizations across nations. Sage publications; 2001.

[72] Hornsey MJ, Harris EA, Bain PG, Fielding KS. Meta-analyses of the determinants and outcomes of belief in climate change. Nat Clim Chang. 2016;6(6):622–6.

[73] Kollmuss A, Agyeman J. Mind the gap: why do people act environmentally and what are the barriers to pro-environmental behavior? Environ Educ Res. 2002;8(3):239–60.

[74] Whitmarsh L, O’Neill S. Green identity, green living? The role of pro-environmental self-identity in determining consistency across diverse pro-environmental behaviours. J Environ Psychol. 2010;30(3):305–14.

[75] Nolan JM, Schultz PW, Cialdini RB, Goldstein NJ, Griskevicius V. Normative social influence is underdetected. Pers Soc Psychol Bull. 2008;34(7):913–23. 18550863 10.1177/0146167208316691

[76] Ferrara, E, Chang, H, Chen, E, Muric, G, Patel, J. Characterizing social media manipulation in the2020 U.S. presidential election. First Monday. 2020;25(11). 10.5210/fm.v25i11.11431.

[77] Matz SC, Kosinski M, Nave G, Stillwell DJ. Psychological targeting as an effective approach to digital mass persuasion. Proc Natl Acad Sci. 2017;114(48):12714–9. 29133409 10.1073/pnas.1710966114 PMC5715760

