# Supplementary material for: The power of loss: message framing, climate anxiety, and engagement in personal carbon trading
Source: BMC Psychol. 2025 Dec 29;13:1381. doi: 10.1186/s40359-025-03713-w (PMC12751754; doi:10.1186/s40359-025-03713-w)
Supplement: Supplementary file 1 — Supplementary Material 1. [file 40359_2025_3713_MOESM1_ESM.docx]

**Appendix A: Specified items of the utilized scale.**

The framing messages is as followed:

*Gain-framed message: By participating in personal carbon trading, you will directly help reduce CO2 emissions. Through your active engagement, air quality is expected to improve within years, creating a healthier environment for future generations.*

*Loss-framed message: By not participating in personal carbon trading, additional CO2 emissions will be generated each year. If we don't act now, air quality may deteriorate within years, threatening the healthy living environment of future generations.*

| Part Ⅰ: Basic information | | | |
| --- | --- | --- | --- |
| Basic information | 1 | BI1 | I understand the basic concept of personal carbon trading. |
|  | 2 | BI2 | I have participated in personal carbon trading related activities. |
|  | 3 | BI3 | Some of my family members or friends have participated in personal carbon trading. |
|  |  |  |  |
| Part Ⅱ: Framing messages | | | |
| Gain-framed information |  |  | By participating in personal carbon trading, you will directly help reduce CO2 emissions. Through your active engagement, air quality is expected to improve within years, creating a healthier environment for future generations. |
| Loss-framed information |  |  | By not participating in personal carbon trading, additional CO2 emissions will be generated each year. If we don't act now, air quality may deteriorate within years, threatening the healthy living environment of future generations. |
|  |  |  |  |
| Part Ⅲ: Main scale | | | |
| Felt responsibility (FR) | 4 | FR1 | I believe participating in personal carbon trading is important for addressing climate change. |
|  | 5 | FR2 | I feel responsible for contributing to carbon emission reduction through personal carbon trading. |
|  | 6 | FR3 | I believe it's important to reduce my carbon footprint through personal carbon trading. |
|  | 7 | FR4 | I feel responsible for taking actions to reduce carbon emissions in my daily life. |
|  | 8 | FR5 | I believe adopting a low-carbon lifestyle is important for environmental protection. |
| Climate change anxiety (CCA) | 9 | CCA1 | Thinking about climate change makes it difficult for me to concentrate. |
|  | 10 | CCA2 | Thinking about climate change makes it difficult for me to sleep. |
|  | 11 | CCA3 | I feel panic about the consequences of climate change. |
|  | 12 | CCA4 | Environmental problems caused by climate change make me feel depressed. |
|  | 13 | CCA5 | I think, "why can't I handle climate change better?" |
|  | 14 | CCA6 | I go away by myself and think about why I feel this way about climate change. |
|  | 15 | CCA7 | I write down my thoughts about climate change and analyze them. |
|  | 16 | CCA8 | I think, "why do I react to climate change this way?" |
|  | 17 | CCA9 | My concerns about climate change make it hard for me to have fun with my family or friends. |
|  | 18 | CCA10 | I have problems balancing my concerns about sustainability with the needs of my family. |
|  | 19 | CCA11 | My concerns about climate change interfere with my ability to get work or school assignments done. |
|  | 20 | CCA12 | My concerns about climate change undermine my ability to work to my potential. |
|  | 21 | CCA13 | My friends say I think about climate change too much. |
| Green self-efficacy (GSE) | 22 | GSE1 | I feel I can succeed in accomplishing environmental ideas. |
|  | 23 | GSE2 | I can achieve most of environmental goals. |
|  | 24 | GSE3 | I feel competent to deal effectively with environmental tasks. |
|  | 25 | GSE4 | I can perform effectively on environmental missions. |
|  | 26 | GSE5 | I can overcome environmental problems. |
|  | 27 | GSE6 | I could find out creative solutions to environmental problems. |
| Voluntary engagement intention towards personal carbon trading (VEI) | 28 | VEI1 | I will often participate in low-carbon activities through the personal carbon trading platform. |
|  | 29 | VEI2 | I will often encourage my family and friends to participate in personal carbon trading. |
|  | 30 | VEI3 | I will often use carbon credits to exchange for goods or services. |
|  | 31 | VEI4 | I am willing to maintain long-term participation in personal carbon trading. |
|  | 32 | VEI5 | I am willing to actively learn about personal carbon trading information. |
|  | 33 | VEI6 | I am willing to make personal carbon trading a part of my daily life. |
|  |  |  |  |
| Part Ⅳ: Descriptive information | | | |
| Descriptive information （DI） | 34 | DI1 | Your gender: Male/ Female |
|  | 35 | DI2 | Your age group: 18-25/ 26-35/ 36-45/ 46-55/ 56 and above |
|  | 36 | DI3 | Your highest education level: High school or below/ College & Bachelor's degree/ Master's degree or above |
|  | 37 | DI4 | Your monthly income range (CNY): 9215 and below/ 9215-20442/ 20443-32195/ 32196-50220/ 50221-95055/ 95055 and above |
